# Multimodal imaging of aberrant macular microvessel crossing the foveal avascular zone in two young adults

**DOI:** 10.1186/s12886-020-01469-y

**Published:** 2020-05-25

**Authors:** Xianming Jiang, Cong Zheng, Fangfang Du, Shibei Ai

**Affiliations:** Department of Ophthalmology, The Seventh Affiliated Hospital of Sun Yat-sen University, 628 Zhenyuan Road, Guangming District, Shenzhen, 518000 P.R. China

**Keywords:** Foveal avascular zone, Aberrant microvessel, Optical coherence tomography angiography, Congenital retinal macrovessel

## Abstract

**Background:**

The traditional view is that there are no vessels in the foveal avascular zone. The two cases we report show microvessels crossing the foveal avascular zone.

**Case presentation:**

A man and a woman, both 25 years old, were both incidentally found on optical coherence tomography angiography (OCTA) to have unilateral aberrant microvessels crossing the foveal avascular zone in their left eyes. Visual acuity was preserved in both patients. The vessel density (VD) and perfusion density (PD) of the eyes with the aberrant microvessels were all higher than those of the contralateral eyes. Nevertheless, measurements of foveal avascular zone (FAZ) dimensions, including its area, perimeter and circularity, were smaller in the left eyes than in the right eyes. No complications were recorded.

**Conclusions:**

To date, aberrant microvessels crossing the foveal avascular zone have not been found to impair visual function. OCTA is a non-invasive and quick method that does not require dilation or the use of fluorescein dye. It is a reliable tool for the detection of aberrant microvessels crossing the foveal avascular zone.

## Background

The fovea is the centre of the macula and is characterized by thinning of the outer nuclear layer. In the foveola, the most central portion of the fovea, the photoreceptors are all cones. The fovea is supplied entirely by the choriocapillaris. The fovea corresponds to the retinal avascular zone viewed on fluorescein angiography [1]. By definition, there are no vessels in the foveal avascular zone. OCTA produces more detailed, higher resolution images of the vasculature than are obtained using conventional dye angiography [2] and is a convenient method for the noninvasive study of the foveal avascular zone. We present two unrelated cases in which unilateral aberrant microvessels crossing the foveal avascular zone were coincidentally detected using OCTA. While there have been many case reports of aberrant macular vessels, there is no clear indication as to whether the aberrant microvessels found in these cases passed through the foveal avascular zone.Both patients were examined at the Department of Ophthalmology of the Seventh Affiliated Hospital Sun Yat-sen University in Shenzhen, China. During routine health checks, on OCTA, a man and woman, both 25 years old, were incidentally found to have unilateral aberrant microvessels crossing the foveal avascular zone in the left eye. We then performed ophthalmological examinations, including optometry, measurement of intraocular pressure with a non-contact tonometer, anterior segment examination, visual field examination (Zeiss, 750i), colour fundus photography (Zeiss, VISUCAM224) and OCTA (Zeiss, Cirrus HD 5000) in both patients.

## Case presentations

### Patient 1

**Fig. 1:**
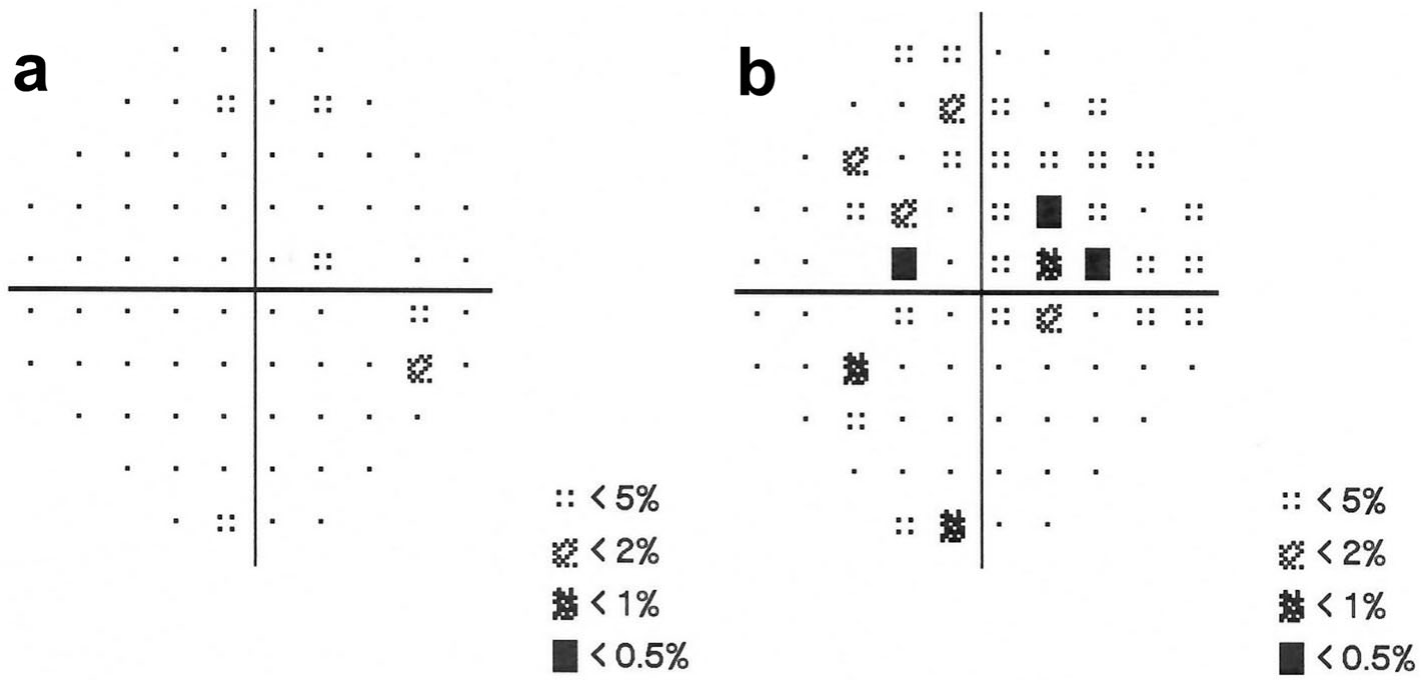
Visual fields (central 30–2 threshold test) in patient 1. **a** Examination of the visual field showing it was normal in the right eye; **b** Examination of the visual field showing a few paracentral scotomas in the left eye

**Fig. 2:**
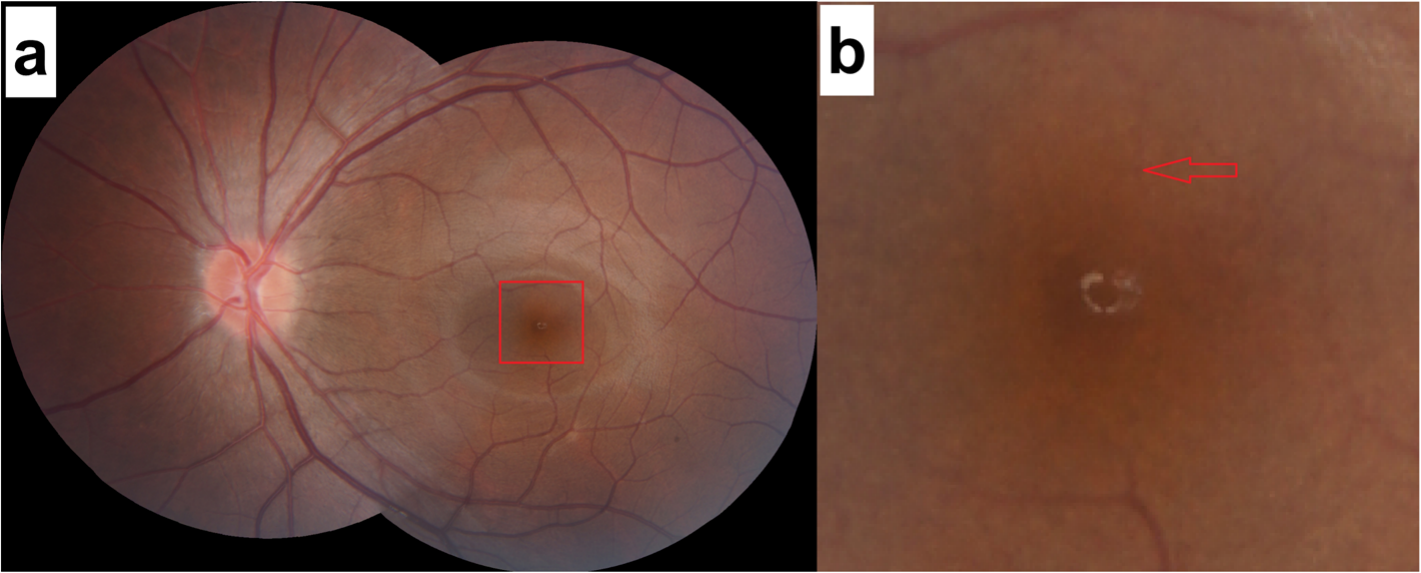
Colour fundus photos of the left eye in patient 1. **a** 45-degree 2-direction fundus photograph. The red frame indicates the position of the amplifier section; **b** Amplifier section. The red arrow shows the location of the aberrant microvessel that crossed the foveal avascular zone

**Fig. 3:**
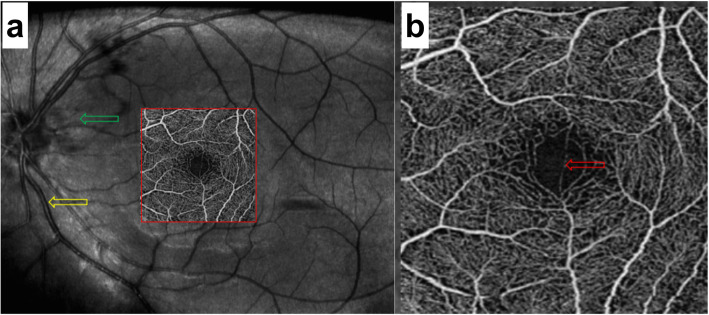
Fundus images of the left eye on OCTA (3 mm × 3 mm scan pattern) in patient 1. **a** The red frame indicates the amplifier section, the green frame indicates the original artery, and the yellow frame indicates the confluent vein. **b** The red arrow reveals the location of the aberrant microvessel that crossed the foveal avascular zone

**Fig. 4:**
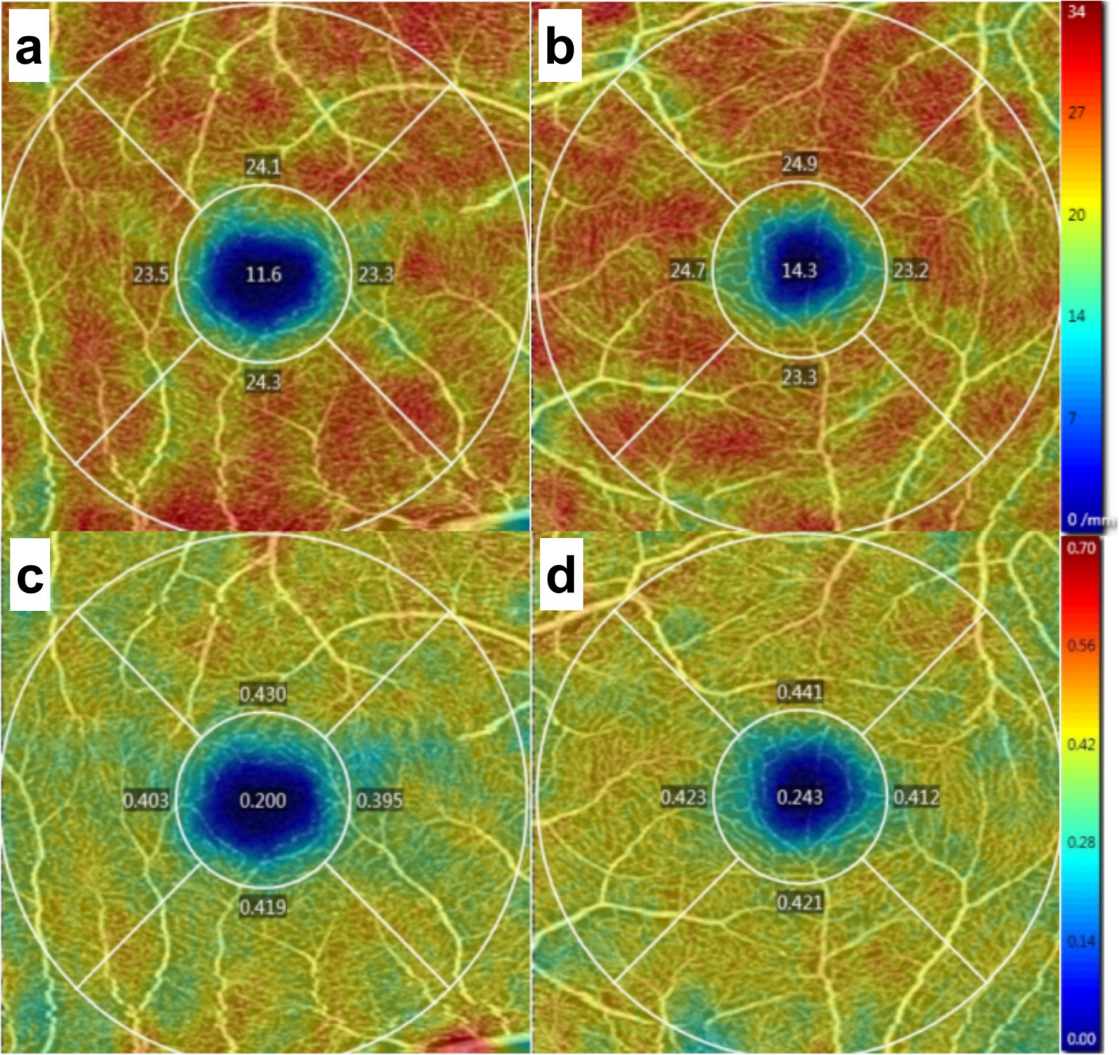
Vessel density and perfusion density on OCTA (3 mm × 3 mm scan pattern) in patient 1. **a** Vessel density of the right eye; **b** Vessel density of the left eye; **c** Perfusion density of the right eye; **d** Perfusion density of the left eye

**Fig. 5:**
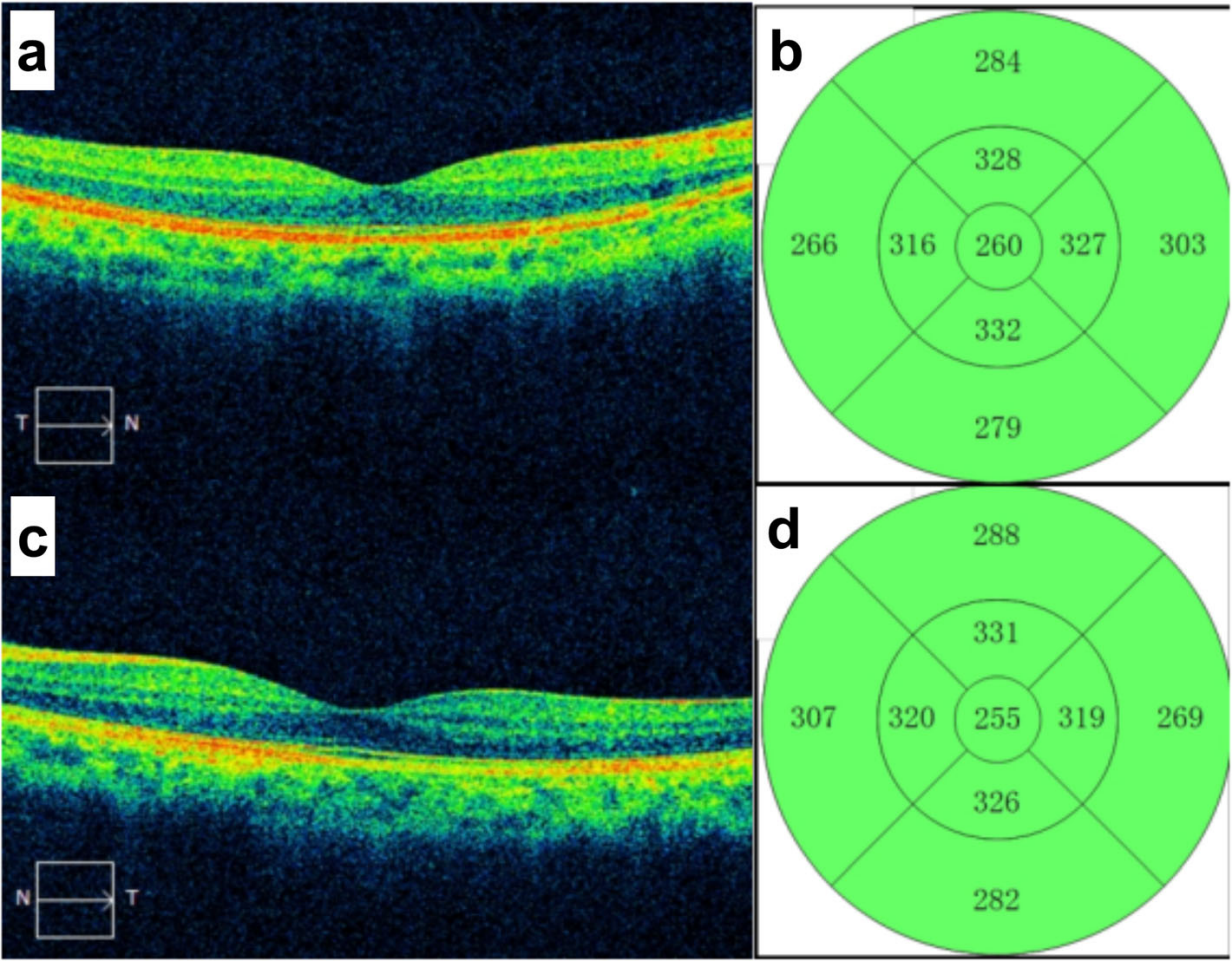
Macular thickness on OCT (512 × 128 scan pattern) in patient 1. **a** Horizontal scan of the right eye; **b** Inner limiting membrane- retinal pigment epithelium (ILM-RPE) thickness of the right eye; **c** Horizontal scan of the left eye; (d) ILM-RPE thickness of the left eye

The first patient was a 25-year-old man with moderate myopia. On ophthalmic examination, his best-corrected visual acuity was 20/20 in both eyes, and the intraocular pressure was within normal limits. The anterior segment examinations performed in both eyes were normal. There were paracentral scotomas in his left eye, and a glaucoma hemifield test (GHT) of the left eye was borderline compared with the GHT of the normal right eye (Fig. 1). It was not easy to find the aberrant microvessel that crossed the foveal avascular zone using colour fundus photography (Fig. 2); however, a fundus image on OCTA clearly revealed the vessel in the left eye (Fig. 3). This aberrant vessel was a small perifoveolar arteriovenous communication. The central retinal artery divides into two branches at the optic disc: the superior artery and the inferior artery. After an extremely short ascending course following its origin, the superior artery separated into a macular branch that then horizontally reached the macula. The abnormal vessel traversed vertically over the entire macula and eventually converged with the branch of the inferior temporal vein. For the central or inner ring, the values for VD and PD obtained in the left eye surpassed those of the opposite eye (Fig. 4, Table 1). Nevertheless, the measurements obtained for FAZ dimensions, including its area, perimeter and circularity, were markedly smaller in the left eye than in the contralateral eye (Table 2). Macular oedema and exudation were not recorded (Fig. 5).

**Table 1:** Vessel density and perfusion density by OCTA in patient 1 (3 mm × 3 mm scan pattern)

Parameter	Right Eye			Left Eye		
	Central	Inner	Full	Central	Inner	Full
Vessel Density (mm^−1^)	11.6	23.8	22.4	14.3	24.0	22.9
Perfusion Density	0.200	0.412	0.388	0.243	0.424	0.404

**Table 2:** Foveal avascular zone by OCTA in patient 1 (3 mm × 3 mm scan pattern)

Parameter	Right Eye			Left Eye		
	Area (mm^2^)	Perimeter (mm)	Circularity	Area (mm^2^)	Perimeter (mm)	Circularity
FAZ	0.25	2.13	0.69	0.18	1.97	0.57

### Patient 2

**Fig. 6:**
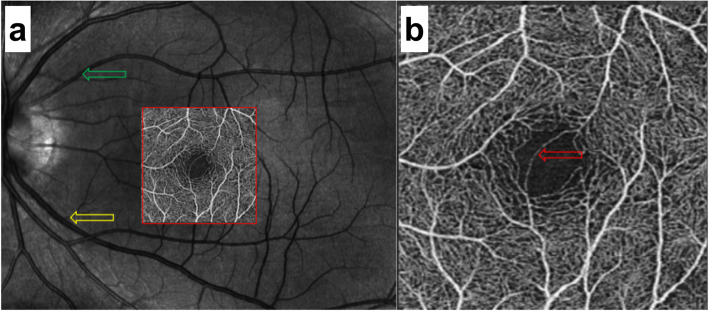
Fundus images of the left eye on OCTA (3 mm × 3 mm scan pattern) in patient 2. **a** The red frame indicates the amplifier section, the green frame indicates the original artery, and the yellow frame indicates the confluent vein. **b** The red arrow reveals the location of the aberrant microvessel that crossed the foveal avascular zone

**Fig. 7:**
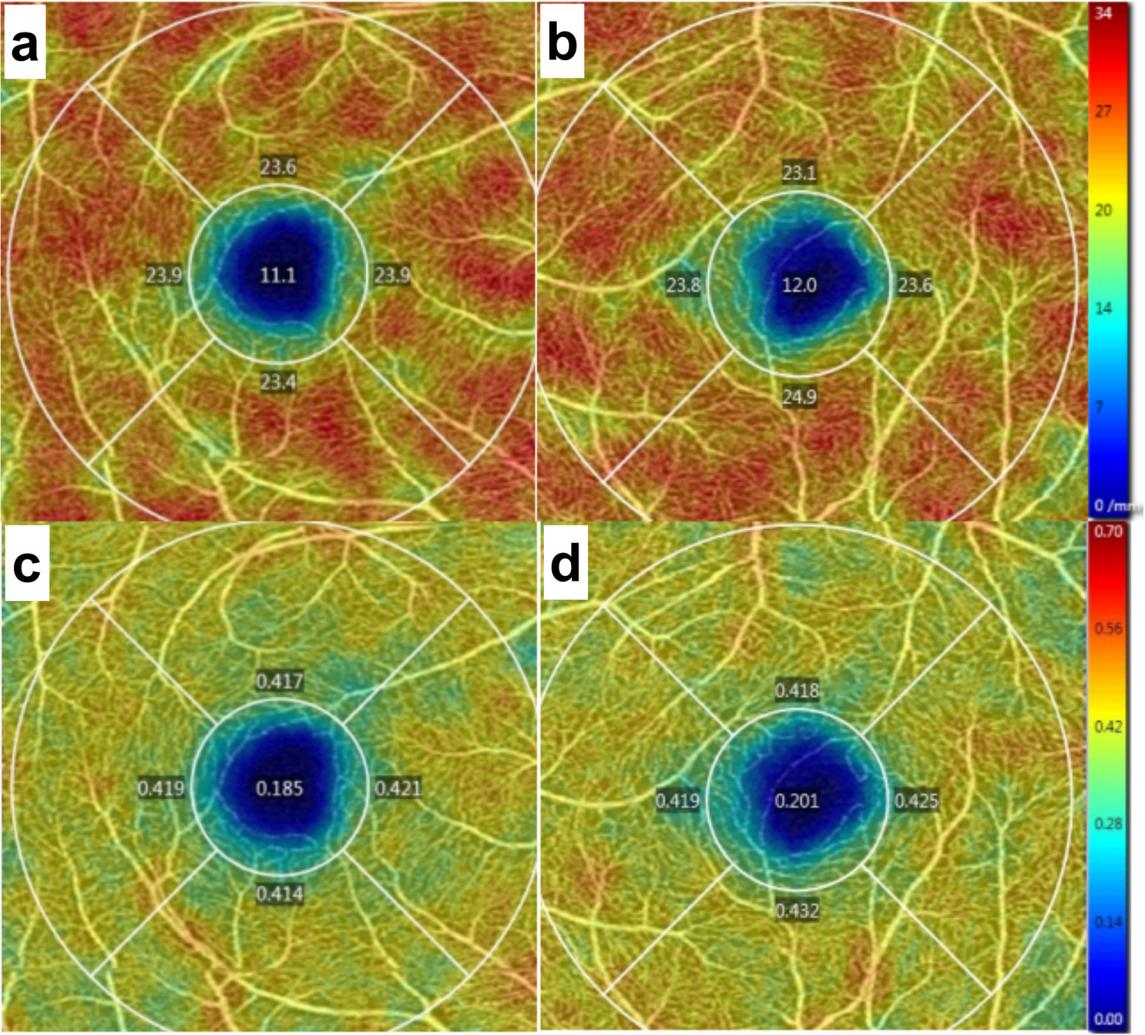
Vessel density and perfusion density on OCTA (3 mm × 3 mm scan pattern) in patient 2. **a** Vessel density of the right eye; **b** Vessel density of the left eye; **c** Perfusion density of the right eye; **d** Perfusion density of the left eye

The second patient was a 25-year-old woman with no past ophthalmic history. Visual acuity was 20/20 without correction in both eyes. Intraocular pressure measurement, anterior segment examination and visual field were normal in both eyes. A fundus image was normal in the right eye, whereas in the left eye, it revealed the presence of an abnormal vessel (Fig. 6). This aberrant microvessel was a communication of the superior temporal artery and the inferior temporal vein. The VD and PD values obtained in the left eye exceeded those obtained in the other eye (Fig. 7, Table 3), whereas the measurements of FAZ dimensions were smaller in the left eye than in the right eye (Table 4). No ocular complications occurred.

**Table 3:** Vessel density and perfusion density by OCTA in patient 2 (3 mm × 3 mm scan pattern)

Parameter	Right Eye			Left Eye		
	Central	Inner	Full	Central	Inner	Full
Vessel Density (mm^−1^)	11.1	23.7	22.3	12.0	23.8	22.5
Perfusion Density	0.185	0.418	0.392	0.201	0.424	0.399

**Table 4:** Foveal avascular zone by OCTA in patient 2 (3 mm × 3 mm scan pattern)

Parameter	Right Eye			Left Eye		
	Area (mm^2^)	Perimeter (mm)	Circularity	Area (mm^2^)	Perimeter (mm)	Circularity
FAZ	0.29	2.14	0.79	0.20	2.01	0.61

## Discussion and conclusions

Many aberrant macular vessels have been reported in the past. Congenital retinal macrovessel is one of the most frequently reported events and was first described in 1869 by Mauthner [3] and named in 1982 by Brown. It refers to one large vessel, most often a vein, that traverses through the central macula and crosses the horizontal raphe [4]. Congenital retinal macrovessel is rare and tends to remain stable. As shown in a five-year follow-up study by K. Petropoulos et al., these anomalous vessels are derived from branches of retinal arteries or veins [5]. Because the majority of sufferers are asymptomatic, congenital retinal macrovessel is usually discovered unexpectedly on physical examinations or in patients with other ophthalmic diseases. In many studies and in normal eyes [6], partial congenital retinal macrovessel can be accompanied by branch retinal artery occlusion [7], retinal deep capillary ischaemia [8], macular retinal cavernous haemangioma [9], retinal arteriolar macroaneurysm [10, 11], retinal peripheral telangiectasia [12], vitreous haemorrhage [13], retinal detachment [14], and even venous malformations of the brain [15]. Archer suggested that retinal arteriovenous communications could be divided into three groups [16]. Congenital retinal macrovessel was part of Group 1, which presents as interposition of an arteriolar or abnormal capillary plexus between the major communicating vessels [16].

Aberrant macular vessels have been reported in the past that present as one large vessel that traverses through the central macula. However, it is not clear whether they cross the FAZ. In contrast to previous reports, the two cases we reported involved microvessels that crossed the foveal avascular zone. We can therefore call them “foveal avascular zone aberrant microvessels” (FAZAMs).

In the past, the detection of aberrant macular vessels depended on fluorescein angiography or colour fundus photography. These examinations require the dilation of the pupils, causing blurred vision. Fluorescein angiography is invasive and a lengthy examination occasionally accompanied with allergic reactions. All of these inconveniences impact screening and follow-up.

Currently, noninvasive and rapid OCTA facilitates examinations. OCTA does not require dilation or fluorescein dye [17] and thus avoids drug-related contraindications. Not only is OCTA accessible for visualization of the retinal vasculature and FAZ, but it also has favourable repeatability and reproducibility [18, 19].

In this study, OCTA revealed the origin and location of aberrant microvessels that crossed the foveal avascular zone and allowed the digitalization and visualization of the vessel density and perfusion density of the retina and measurements of foveal avascular zone dimensions and the macular thickness that excluded the ocular complications involving macular oedema and exudation. Our description of aberrant microvessels crossing the foveal avascular zone was comprehensive and systematic. Most importantly, the VD and PD were higher in the eyes with aberrant microvessels crossing the foveal avascular zone than in those the contralateral eyes, whereas the measurements of FAZ dimensions were lower. This was probably because the arteriovenous anastomosis above the macular area increased the arterial supply of the retina and damaged the circular configuration of the FAZ. More clinical data need to be acquired to support this opinion.

We show that foveal avascular zone aberrant microvessels can cross the FAZ; however, their visual function was not affected. The possible reasons are as follows: the diameter of the aberrant microvessel is too small to affect visual function, or the vision changes caused by the abnormal blood vessels were too small to be detected by existing instruments. Aberrant blood vessels are congenital, and functional compensation may occur during macular development. Of course, conclusions regarding the follow-up effects of visual function require further observation.

In conclusion, aberrant microvessels were found in the foveal avascular zone, which should not contain vessels; however, no visual function impairment was found. OCTA is noninvasive and rapid, does not require dilation or fluorescein dye, and is a reliable tool to observe aberrant microvessels crossing the foveal avascular zone.

## Data Availability

The datasets used and analysed during the current study are available from the corresponding author on a reasonable request.

## Abbreviations

OCTA: Optical coherence tomography angiography
VD: Vessel density
PD: Perfusion density
FAZ: Foveal avascular zone
GHT: Glaucoma hemifield test
ILM-RPE: Inner limiting membrane- retinal pigment epithelium
